# 

**Published:** 1924-05

**Authors:** 


### PUBLIC HEALTH IN PARLIAMENT.

The following have been selected from recent answers to questions in Parliament as being of general interest.

### NATIONAL HEALTH INSURANCE.

Lapsed Policies.—Under the Prolongation of Insurance Act, 1921, any insured person who had been in regular employment prior to the present period of industrial depression is safeguarded from lapsing from insurance by reason of prolonged unemployment.

Sickness Benefit.—Under the Act, sickness benefit is only payable from the fourth day of incapacity, but any Approved Society which is found on valuation to have a disposable surplus may provide for the payment of sickness benefit from the first day. Out of 6,659 Societies and branches in England which had a disposable surplus on the first valuation, only 66 elected to adopt this form of additional benefit. An increase of the weekly contribution would be necessary in order to place Societies in a position to pay benefit from the first day otherwise than by recourse to their surplus funds.

Cost of Administration.—The amounts expended on administration of National Health Insurance in England and Wales in the year 1922 were as follows:—Approved Societies, £2,913,000; Ministry of Health and other Government Departments, £1,010,000; Insurance Committees, £378,000.

Administration Allowance.—There is no evidence that the work of Approved Societies has been seriously affected as a result of the reduction in the maximum allowance for expenditure on administration, and there is no reason to think that any increase of the present sum is required in order that Societies should adequately perform their functions.

Health Insurance and Workmen's Compensation.—The question of amalgamating the sickness insurance scheme with the existing statutory provision for compensation for workmen in the case of accidents and industrial disease was fully considered in 1920 by the Holman Gregory Committee, who came unanimously to the conclusion that such a course is not practicable. In the course of ordinary business during the Session, opportunity might be taken to have this subject discussed.

### TUBERCULOSIS.

After-Care Colonies.—There are at present three after-care colonies accommodating some 100 married men and their families and 80 single men. I am aware also of two non-residential employment centres and one centre partly non-residential. I am advised that establishments of this kind must be regarded as still in the experimental stage, though the results so far obtained are hopeful.

Village Settlements.—The village settlements for tuberculous ex-Service men are at Papworth and Preston Hall, and £10,000 has been allotted to each for this purpose. The Joint Finance Committee of the British Red Cross Society and the Order of St. John of Jerusalem have voted the sum of £10,000 to the East Lancashire Tuberculosis Colony in order that cottages may be provided therefor tuberculous ex-Service men. A report has been called for as to the present position at Barrowmore and the need for any further development.

### ANSWERS TO CORRESPONDENTS.

R. S.—The Weir Hospital, Grove Road, Balham, S.W., would probably be the most convenient. Admission is by letter of recommendation.

John.—The official opening of the King Edward VII. Memorial Sanatorium at Hertford Hill, Warwick, will take place this year. It was opened informally last October.

M.—Such a pamphlet was published last year by the Association of Headmistresses and entitled "Nursing as a Profession." It is intended for the educated girl.

Hospital Secretary.—The charge for outside sleeping accommodation for the hospital staff should be entered under: (c) Rent, Rates and Taxes; (l) Rent. It should not be placed under Extraordinary Expenditure. The item, Rent, Rates and Taxes, is not included in the calculation of cost per occupied bed.

Queen's Nurses.—Yes; to become a Queen's Nurse you must have three years' general training as well as the six months' specialised training. If you have any other qualifications so much the better.

### RECENT APPOINTMENTS.

### Medical Officers, &c.

Braithwaite, Joseph, M.B., Ch.B.; Junior Assistant M.O. Royal Earlswood Institution (Edinburgh University).

Buckell, A. C. M., M.B., B.S., D.P.H.; City Bacteriologist, Bradford (£750-£1,000).

Butcher, W. H., M.A., M.D., D.P.H.; Assistant M.O., Somerset.

Dunscombe, Nicholas D., M.A., M.B., D.P.H.; Assistant M.O., Somerset.

Fairweather, J. W. C., M.B., Ch.B.; M.O.H., Fordoun.

Geffen, Dennis H., M.R.C.S., L.R.C.P.; M.O.H., Finsbury.

Herbst, Margaret Barbara, M.B., B.S.; Assistant School M.O., Gateshead (£600).

M'Gonigle, G.C.M., M.D., D.P.H; M.O.H., Stockton.

Newman, R. H., L.R.C.P., L.R.C.S.(Ireland); M.O.H., Stannington.

Pattison, C. Lee, M.B., B.S., L.R.C.P., F.R.C.S; Consulting Surgeon, Tuberculosis Department, Rotherham Corporation.

Reid, William, M.B., Ch.B., Senior Assistant M.O., County Mental Hospital, Burntwood, Lichfield; Medical Superintendent, County Mental Hospital, Burntwood.

Robertson, Alexander, M.B., Ch.B., D.P.H.; M.O.H., Elgin.

Sergeant Bertram, M.B., B.S.; Assistant School M.O., Gateshead (£600).

Stirling, J. A., M.B., Ch.B.; Assistant M.O., West Hartlepool (£600).

### Public Health and Hospital Appointments.

Coggins, M. K., Matron, West London Hospital; Matron, Sheffield Royal Hospital.

Cordell, T. W.; Second Sanitary Inspector, Hailsham (£160).

Gillett-Gatty, E. K.; Health Visitor, New Windsor.

Hodgins, F. M., R. R. C.; Matron-in-Chief, Queen Alexandra's Imperial Military Nursing Service.

Jones, Amy, Sister in Charge Caerphilly Hospital; Matron, Harwich and District Cottage Hospital.

Kirkpatrick, Jessie Munro; Matron, District and General Hospital, Kettering.

McNicol, Elizabeth; Matron, Oxford County and City Mental Hospital.

Milner, Louie; School Nurse, Dewsbury.

Milton, Dorothy May, Assistant Matron, Warneford, Leamington and South Warwickshire General Hospital; Matron, Royal Cornwall Infirmary, Truro.

Moggach, Gertrude; Matron, Bury Infirmary.

Muirhead, Katherine; Matron, Cumberland Infirmary, Carlisle.

Phelp, Mrs.; Superintendent, Fife County Nursing Association, Queen Victoria's Jubilee Institute

Robus, Mabel F.; Health Visitor, Deptford.

Thorpe, E. K.; Matron, Kidderminster Infirmary.

Timpson, Ethel; Matron, Caterham Cottage Hospital.

Tonz, M.; Matron, Paulton Memorial Hospital, Somerset.

Vaughan-Winters, M., Matron, Royal Cornwall Infirmary, Truro; Matron, South London Hospital for Women.

Wade, K. E.; Tuberculosis Nurse, Barnsley.

Wilton, John William: Senior Sanitary Inspector, Hailsham (£420).

[By an error that we much regret it was stated in last month's issue that Miss Williamina Thaw was appointed Superintendent of the Queen Victoria Jubilee Nurses' Institute at Greenock, instead of Assistant Superintendent. Miss Mary Bruce is the Superintendent.]

### A Birmingham Cancer Inquiry.

With the object of inquiring into the prevalence of cancer, the facilities for the treatment of cancer (both operative and remedial), and allied matters, the Birmingham City Council has appointed a sub-committee of its health committee of which Councillor Miss Bartlett is chairman. Birmingham's innovation has resulted largely from the efforts and recommendations of Councillor Dr. J. Hall Edwards.

### Managerial Notices.

*All letters on Business matters must be addressed to the Manager, "The Hospital and Health Review," 28 and 29 Southampton Street, London, W.C. 2.*

RATES FOR SUBSCRIPTION (payable in advance).

| | | |
|---|---|---|
| Three Months (including Postage) | | 1s. 9d. |
| Six Months | do. | 3s. 6d. |
| Twelve Months | do. | 7s. 0d. |

**It is especially requested that remittances be made by Cheque or Postal Order and not Stamps.**

Subscriptions, which may commence at any time, are payable in advance. Cheques and Post-Office Orders should be crossed Westminster Bank, Covent Garden Branch, and made payable to the Manager of THE HOSPITAL AND HEALTH REVIEW.

***Advertisements.*** Scale of charges and particulars of special positions may be obtained on application to the Manager.

*SUBSCRIPTION FORM.*

*Please send me* THE HOSPITAL AND HEALTH REVIEW *for*............ *months, commencing with your issue of* ............, *for which please find enclosed* .......

*Name* ..............................

*Address* ..............................

..............................

*Date* ..............................

*To*...... ..............................*Newsagent*

